# Association of Genetic Loci with Blood Lipids in the Chinese Population

**DOI:** 10.1371/journal.pone.0027305

**Published:** 2011-11-03

**Authors:** Zhou Zhang, Liming Tao, Zhuo Chen, Daizhan Zhou, Mengyuan Kan, Di Zhang, Can Li, Lin He, Yun Liu

**Affiliations:** 1 Institute for Nutritional Sciences, Shanghai Institutes for Biological Sciences, Chinese Academy of Sciences, Graduate School of the Chinese Academy of Sciences, Shanghai, People's Republic of China; 2 Institutes of Biomedical Sciences, Fudan University, Shanghai, People's Republic of China; 3 Bio-X Center, Key Laboratory of Developmental Genetics and Neuropsychiatric Diseases (Ministry of Education), Shanghai Jiao Tong University, Shanghai, People's Republic of China; California State University Fullerton, United States of America

## Abstract

**Background:**

Recent genome-wide association (GWA) studies have identified a number of novel genetic determinants of blood lipid concentrations in Europeans. However, it is still unclear whether these loci identified in the Caucasian GWA studies also exert the same effect on lipid concentrations in the Chinese population.

**Methods and Results:**

We conducted a replication study assessing associations between SNPs at 15 loci and blood lipid and lipoprotein concentrations in two Chinese cohorts, comprising 2533 and 2105 individuals respectively. SNPs in *APO(A1/C3/A4/A5), TIMD4-HAVCR1, DOCK7, TRIB1, ABCA1,* and *TOMM40-APOE* showed strong associations with at least one lipids trait, and rs174546 in *FADS1/2/3* showed modest association with triglyceride in the Chinese population.

**Conclusions:**

We successfully replicated 7 loci associated plasma lipid concentrations in the Chinese population. Our study confirmed the implication of *APO(A1/C3/A4/A5), TOMM40-APOE, ABCA1, DOCK7, TIMD4-HAVCR1, TRIB1* and *FADS1/2* in plasma lipid and lipoprotein concentrations in Chinese population.

## Introduction

Plasma lipids and lipoproteins concentrations are important risk factors for atherosclerosis and related vascular diseases [1]. Twin and family studies suggest that about 50% of the variation in plasma lipid and lipoprotein levels is genetically determined [2].

A search for the genetic contributions to variation in plasma lipid and lipoprotein levels has been ongoing for several decades [3]. Since 2007, genome-wide association (GWA) studies have obtained great success and have implicated common variants in numerous loci and genes as being the genetic influences underlying lipid and lipoprotein levels [4], [5], [6], [7], [8], [9], [10], [11]. Kathiresan's group [12] recently performed a comprehensive meta-analysis of the GWA studies and identified 95 loci significantly associated with blood lipids. These associations were primarily found in European ancestry. Following studies successfully replicated most of these loci in East Asian population [12], [13], [14], [15], [16], [17]. However, some of the loci showed no significant association with lipid and lipoprotein levels in Asian population, including the *MAFB*, *NCAN/CLIP2/PBX4* and *MVK/MMAB* loci [12], [14], [17]. It is therefore important to confirm whether known loci have consistent effects across ethnic groups.

The purpose of this study was to replicate the previously reported genetic loci in the Chinese population. We firstly evaluated association between lipid levels and 15 loci selected from the three recent GWAS reports [4], [7], [9] in Cohort1, comprising 2, 533 Chinese individuals, and selected 10 out of the 15 loci on the basis of the strength of statistical evidence. We then tested association of the 10 loci with lipid traits in Cohort2, comprising 2,105 individuals, to confirm the findings in Cohort1. Finally, we combined the two cohorts results together, since both the two cohort were from Shanghai, with similar genetic background.

Clinically, the most important plasma lipids and lipoproteins are triglycerides (TG), total cholesterol (TC), high density lipoprotein (HDL) cholesterol and low density lipoprotein (LDL) cholesterol. Several studies have suggested that the lipid ratio (TC/HDL-C) has greater independent predictive value for coronary heart disease (CHD) and cardiovascular events than either total cholesterol or LDL cholesterol levels [3], [18]. We therefore focused on five lipid traits: TG, TC, HDL cholesterol, LDL cholesterol and the TC/HDL ratio in this study.

## Methods

### Ethics Statement

The ethics committee of the Shanghai Institute for Biological Sciences approved this study. Written consents were given by the patients.

### Participants

Participants in the present study comprised two groups, Cohort1 and Cohort2. Cohort1 was primarily designed for a case-control study of type 2 diabetes (including 1,360 non-type 2 diabetes controls and 1,173 type 2 diabetes patients). Cohort2 was a community-based prospective epidemiologic cohort of 2,105 subjects. Individuals known to be on lipid-lowering therapy were excluded. Both cohorts were recruited from Shanghai, China. The characteristics of participants are summarized in Table 1.

**Table 1:** Participant characteristics.

Variable	Cohort1	Cohort2
	n = 2533	n = 2105
Female gender (%)	65.3	68.7
Age(years)	61±9	59±10
Height(cm)	159.5±7.7	160.9±8.0
BMI(kg/m^2^)	25.0±3.4	24.7±3.3
TC(mmol/l)	4.97±0.95	4.53±0.91
TG(mmol/l)	1.30(0.94∼1.86)	1.41(1.01∼2.06)
HDL-C(mmol/l)	1.19±0.30	1.24±0.31
LDL-C(mmol/l)	3.02±0.79	2.84±0.75
TC/HDL-C	4.26(3.51∼5.04)	3.70(3.09∼4.48)
Individuals with T2D (%)	1173(46.3)	197(9.4)

Data are shown as mean±standard deviation, or median (25% quantile∼75% quantile).

T2D, type 2 diabetes.

For all individuals, height, weight, hip and waist circumference and blood pressure were measured by trained medical professionals using a standardized protocol. Body mass index (BMI) was calculated as weight (kg)/[height (m)]^2^. Blood samples were collected after an overnight fast. Total cholesterol, HDL cholesterol, LDL cholesterol, triglycerides, and fasting plasma glucose (FPG) were measured enzymatically according to standard methods on the Roche modular P800 autoanalyzer (Roche, Mannheim, Germany) with the appropriate reagents (Roche Diagnostics CmbH, Mannheim, Germany).

### Selection of candidate variants

We selected 15 out of 55 single nucleotide polymorphisms (SNPs) that achieved genome-wide statistical significance in three recently published GWA studies [4], [7], [9]. Three criteria were adopted to choose SNPs:

1. Only one lead SNP was selected for each locus. For instance, three GWAS reported seven SNPs in the *FADS1/2/3* cluster, including rs174570, [4] rs174537, rs102275, rs174556, rs1535, rs174546, [9] and rs174547 [7]. Considering all were in a region with extremely high degree of linkage disequilibrium (D' = 1.0, r^2^ = 1.0, The International HapMap Project), we selected one variant, rs174546, to represent this region.
2. We selected only those SNPs with a minor allele frequency higher than 5% in Chinese population to ensure that this study had enough statistical power.
3. Loci had been studied in Chinese population [13] were excluded.

### Genotyping

High-molecular-weight genomic DNA was prepared from venous blood using the QuickGene 610 L Automatic DNA/RNA Extraction System (Fijifilm, Tokyo, Japan). All genotyping experiments were done using TaqMan technology on an ABI7900 system (Applied Biosystems, Foster City, California). The standard 5 µl polymerase chain reaction (PCR) reactions were carried out using TaqMan Universal PCR Master Mix reagent kits under the guidelines provided. Genotype data were obtained in 97.5% of the DNA samples. Replicate quality control samples (5% samples) were included and genotyped with 100% concordance.

### Statistical analyses

SHEsis [19] was used to perform the Hardy-Weinberg Equilibrium (HWE) test. We assumed an additive model of inheritance, and conducted multiple linear regressions to assess the effect of the number of the specified allele of each SNP on five traits—concentrations of TC, TG, LDL cholesterol, HDL cholesterol and the TC/HDL ratio. Age, gender, body mass index (BMI), and type 2 diabetes status were included in the multiple linear regression models as covariant. Plasma TG and TC/HDL-C were logarithmically transformed before linear regression due to skewed distributions. We did not exclude the type 2 diabetes subjects, in line with previous genome-wide studies [6], [10]. Considering the potential correlation between diabetes and lipid levels, we included the diabetes status in the association model as a covariant. Multiple testing corrections were performed in stage 2 and combined analysis, *P*<0.001 was considered as significant, given there were 10 SNPs and 5 traits analyzed.

## Results

### Stage 1 replication

15 SNPs in 15 loci were genotyped in Cohort 1 comprising 2533 individuals at stage 1. Genotype distribution of each SNP did not deviate from Hardy-Weinberg equilibrium at the 5% level. Results of the multiple linear regression analysis adjusted for age, gender, BMI, and type 2 diabetes status are shown in Table 2.

**Table 2:** Association of SNPs with TC, TG, HDL, LDL, and TC/HDL in Cohort1.

							TC		log(TG)		HDL		LDL		log(TC/HDL)	
SNP	Chr.	Position(Mb)	Nearest gene(s)	Traits^a^	Alleles^b^	MAF^c^	beta^d^	*P* value^e^	beta^d^	*P* value^e^	beta^d^	*P* value^e^	beta^d^	*P* value^e^	beta^d^	*P* value^e^
rs10903129	1	25.64	*TMEM57*	TC,LDL	A/G	0.3	0.04	0.21	−0.02	0.22	0.01	0.33	0.04	0.1	0	0.78
rs10889353	1	62.89	*DOCK7*	TC,TG	A/C	0.19	−0.1	**3.2E-03**	−0.03	0.07	−0.01	0.55	−0.05	0.1	−0.02	0.1
rs1501908	5	156.33	*TIMD4-HAVCR1*	LDL	C/G	0.27	−0.07	**0.02**	−0.03	**0.04**	0.01	0.13	−0.05	**0.03**	−0.03	**5.1E-03**
rs12670798	7	21.57	*DNAH11*	TC,LDL	C/T	0.5	−0.02	0.48	−0.01	0.37	0.01	0.14	−0.01	0.69	−0.01	0.092
rs2954029	8	126.56	*TRIB1*	TC,TG,LDL	T/A	0.42	0.09	**4.0E-04**	0.04	**3.1E-03**	−0.01	0.34	0.05	**0.01**	0.03	**2.0E-03**
rs1883025	9	106.7	*ABCA1*	HDL	C/T	0.22	−0.1	**1.3E-03**	−0.04	**0.01**	−0.02	**0.02**	−0.03	0.2	0	0.87
rs964184	11	116.15	*APO(A1/C3/A4/A5)*	TG,HDL	C/G	0.22	0.02	0.57	0.16	**1.1E-20**	−0.05	**1.0E-07**	−0.1	**3.6E-04**	0.05	**3.6E-07**
rs7120118	11	47.24	*NR1H3*	HDL	C/T	0.25	0	0.94	0.01	0.64	0	0.76	0.01	0.73	0	0.87
rs174546	11	61.33	*FADS1-FADS2*	TC,LDL	C/T	0.41	−0.02	0.39	0.02	0.14	0	0.83	0.02	0.43	0	0.59
rs2338104	12	108.38	*MMAB-MVK*	HDL	C/G	0.37	0.06	**0.03**	0.02	0.16	0	0.91	0.04	0.1	0.01	0.17
rs2650000	12	119.87	*HNF1A*	LDL	C/A	0.47	0.07	**0.01**	0.03	**0.03**	0.01	0.54	0.02	0.3	0.01	0.49
rs4939883	18	45.42	*LIPG*	HDL	C/T	0.17	−0.05	0.12	−0.01	0.62	−0.02	0.08	−0.03	0.35	0	0.91
rs2304130	19	19.65	*NCAN*	TC,LDL	A/G	0.14	0.01	0.79	0.02	0.26	−0.01	0.66	−0.01	0.71	0.01	0.45
rs157580	19	50.09	*TOMM40-APOE*	TC,TG,LDL	G/A	0.44	−0.06	**0.02**	0.03	**0.03**	−0.01	0.34	−0.11	**2.7E-07**	−0.01	0.52
rs6102059	20	38.66	*MAFB*	LDL	T/C	0.44	0.05	0.09	0	0.74	0	0.94	0.03	0.27	0.01	0.16

a. Traits reported as associated with loci in previous GWA studies.

b. Alleles are shown as major allele/minor allele.

c. MAF, minor allele frequency estimated from the genotyped data of Cohort1.

d. beta, the coefficient assessed using additive model of the minor allele.

e. Results with P value<0.05 are shown in boldface.

Of the 15 SNPs tested, 8 SNPs including rs10889353 in *DOCK7*, rs1501908 in *TIMD4-HAVCR1*, rs2954029 in *TRIB1*, rs1883025 in *ABCA1*, rs964184 in *APO(A1/C3/A4/A5)*, rs2338104 in *MMAB-MVK*, rs2650000 in *HNF1A*, and rs157580 in *TOMM40-APOE*, showed significant association (P<0.05) with at least one lipid level trait.

However, 7 other loci including rs10903129 located in *TMEM57,* rs12670798 in *DNAH11*, rs4936883 in *LIPG*, rs2304130 in *NCAN*, rs7120118 in *NR1H3*, rs6102059 in *MAFB*, and rs174546 in *FADS1/2*, which had previously been reported to be associated with plasma lipid levels in European ancestry, did not show evidence for association with any of the five lipid traits in the our Chinese sample. Given that the *FADS1/2/3* cluster was reported to be associated with lipid concentrations in all of the three GWAS papers, we included this locus in the stage 2 replication. Rs6102059 in *MAFB* showed a relatively low *P* value with TC (*P*<0.09), therefore was also included.

### Stage 2 replication

The 10 selected SNPs were subsequently genotyped in Cohort2, and 4 of them showed significant association with lipid traits after multiple testing correction (*P*<0.001), including variants in *TIMD4-HAVCR1, TRIB1, ABCA1* and *APO(A1/C3/A4/A5)* (Table 3).

**Table 3:** Association of SNPs with TC, TG, HDL, LDL, and TC/HDL in Cohort2.

							TC		log(TG)		HDL		LDL		log(TC/HDL)	
SNP	Chr.	Position(Mb)	Nearest gene(s)	Traits^a^	Alleles^b^	MAF^c^	beta^d^	*P* value^e^	beta^d^	*P* value^e^	beta^d^	*P* value^e^	beta^d^	*P* value^e^	beta^d^	*P* value^e^
rs10889353	1	62.89	*DOCK7*	TC,TG	A/C	0.19	−0.06	0.07	−0.07	1.5E-03	0.00	0.99	−0.01	0.75	−0.01	0.20
rs1501908	5	156.33	*TIMD4-HAVCR1*	LDL	C/G	0.26	−0.07	0.04	−0.05	0.01	0.02	0.10	−0.05	0.06	−0.03	**9.2E-04**
rs2954029	8	126.56	*TRIB1*	TC,TG,LDL	T/A	0.42	0.08	5.0E-03	0.07	**8.7E-06**	−0.01	0.14	0.06	0.02	0.03	**8.4E-04**
rs1883025	9	106.7	*ABCA1*	HDL	C/T	0.2	−0.06	0.10	−0.03	0.11	−0.05	**5.7E-05**	0.00	0.88	0.02	0.02
rs964184	11	116.15	*APO(A1/C3/A4/A5)*	TG,HDL	C/G	0.23	−0.02	0.50	0.12	**7.2E-10**	−0.05	**3.7E-05**	−0.08	4.9E-03	0.03	**8.2E-04**
rs174546	11	61.33	*FADS1-FADS2*	TC,LDL	C/T	0.42	−0.03	0.27	0.04	0.03	−0.01	0.18	−0.03	0.28	0.00	0.57
rs2338104	12	108.38	*MMAB-MVK*	HDL	C/G	0.35	−0.04	0.21	−0.01	0.66	0.00	0.83	−0.02	0.49	−0.01	0.51
rs2650000	12	119.87	*HNF1A*	LDL	C/A	0.48	−0.04	0.12	−0.02	0.16	0.00	0.98	−0.01	0.59	−0.01	0.23
rs157580	19	50.09	*TOMM40-APOE*	TC,TG,LDL	G/A	0.45	−0.03	0.21	0.02	0.25	0.01	0.45	−0.06	8.5E-03	−0.01	0.11
rs6102059	20	38.66	*MAFB*	LDL	T/C	0.44	0.02	0.40	0.02	0.33	0.01	0.27	0.00	0.85	0.00	0.71

a. Traits reported as associated with loci in previous GWA studies.

b. Alleles are shown as major allele/minor allele.

c. MAF, minor allele frequency estimated from the genotyped data of Cohort1.

d. beta, the coefficient assessed using additive model of the minor allele.

e. Results with P value<0.001 are shown in boldface.

### Combined analysis

Considering the two cohorts were from the same place, we combined the two cohorts together to enhance the statistic power (Table 4). We found three variants, including rs10889353 in *DOCK7* (Combined *P*∼6.5×10^−4^), rs2954029 in *TRIB1* (Combined *P*∼5.8×10^−6^) and rs1883025 in *ABCA1* (Combined *P*∼4.0×10^−4^), associated with total cholesterol concentration. Three variants, including rs10889353 in *DOCK7* (Combined *P*∼5.9×10^−4^), rs2954029 in *TRIB1* (Combined *P*∼2.3×10^−7^) and rs964184 in *APO(A1/C3/A4/A5)* (Combined *P*∼2.8×10^−28^), showed significant association with triglyceride concentrations. Two variants including rs1883025 in *ABCA1* (Combined *P*∼2.0×10^−5^) and rs964184 in *APO(A1/C3/A4/A5)* (Combined *P*∼3.0×10^−11^), showed association with HDL cholesterol concentrations. Three variants including rs2954029 in *TRIB1* (Combined *P*∼7.1×10^−4^), rs964184 in *APO(A1/C3/A4/A5)* (Combined *P*∼4.6×10^−6^) and rs157580 in *TOMM40-APOE* (Combined *P*∼2.0×10^−8^) showed significant association with LDL cholesterol concentrations. Three variants including rs1501908 in *TIMD4-HAVCR1* (Combined P∼1.9×10^−5^), rs2954029 in *TRIB1* (Combined *P*∼6.0×10^−6^) and rs964184 in *APO(A1/C3/A4/A5)* (Combined *P*∼2.5×10^−9^) showed association with the ratio of total cholesterol to HDL cholesterol.

**Table 4:** Association of SNPs with TC, TG, HDL, LDL, and TC/HDL in Combined Cohort.

							TC		log(TG)		HDL		LDL		log(TC/HDL)	
SNP	Chr.	Position(Mb)	Nearest gene(s)	Traits^a^	Alleles^b^	MAF^c^	beta^d^	*P* value^e^	beta^d^	*P* value^e^	beta^d^	*P* value^e^	beta^d^	*P* value^e^	beta^d^	*P* value^e^
rs10889353	1	62.89	*DOCK7*	TC,TG	A/C	0.19	−0.08	**6.5E-04**	−0.05	**5.9E-04**	0.00	0.74	−0.03	0.15	−0.02	0.03
rs1501908	5	156.33	*TIMD4-HAVCR1*	LDL	C/G	0.27	−0.07	1.5E-03	−0.04	2.2E-03	0.02	0.03	−0.05	3.9E-03	−0.03	**1.9E-05**
rs2954029	8	126.56	*TRIB1*	TC,TG,LDL	T/A	0.42	0.09	**5.8E-06**	0.06	**2.3E-07**	−0.01	0.09	0.05	**7.1E-04**	0.03	**6.0E-06**
rs1883025	9	106.7	*ABCA1*	HDL	C/T	0.22	−0.08	**4.0E-04**	−0.04	3.6E-03	−0.03	**2.0E-05**	−0.02	0.35	0.01	0.23
rs964184	11	116.15	*APO(A1/C3/A4/A5)*	TG,HDL	C/G	0.22	0.00	0.95	0.14	**2.8E-28**	−0.05	**3.0E-11**	−0.09	**4.6E-06**	0.04	**2.5E-09**
rs174546	11	61.33	*FADS1-FADS2*	TC,LDL	C/T	0.41	−0.03	0.17	0.03	9.9E-03	−0.01	0.31	0.00	0.92	0.00	0.92
rs2338104	12	108.38	*MMAB-MVK*	HDL	C/G	0.37	0.02	0.42	0.01	0.50	0.00	0.86	0.01	0.41	0.00	0.55
rs2650000	12	119.87	*HNF1A*	LDL	C/A	0.47	0.02	0.37	0.01	0.48	0.00	0.58	0.01	0.69	0.00	0.75
rs157580	19	50.09	*TOMM40-APOE*	TC,TG,LDL	G/A	0.44	−0.05	9.3E-03	0.03	0.01	0.00	0.81	−0.09	**2.0E-08**	−0.01	0.14
rs6102059	20	38.66	*MAFB*	LDL	T/C	0.44	0.04	0.07	0.00	0.68	0.00	0.51	0.01	0.45	0.01	0.37

a. Traits reported as associated with loci in previous GWA studies.

b. Alleles are shown as major allele/minor allele.

c. MAF, minor allele frequency estimated from the genotyped data of Cohort1.

d. beta, the coefficient assessed using additive model of the minor allele.

e. Results with P value<0.001 are shown in boldface.

We found marginal association between rs174546 in the *FADS1/2/3* and triglycerides (Combined *P*<0.01) (Table 4). We found no significant association between rs2338104 in *MMAN-MVK*, rs2650000 in *HNF1A* or rs6102059 in *MAFB* and plasma lipid levels either in Cohort2 or in the combined Cohorts.

## Discussion

In this study, we investigated whether the results of three independent genome-wide European association studies on plasma lipid and lipoprotein levels were replicatable in the Chinese population. Of the 15 loci selected from the European GWAS reports, 7 loci were successfully replicated.

The most significant association was found between rs964184 in the *APO(A1/C3/A4/A5)* cluster and triglycerides (Combined *P*∼2.8×10^−28^). This variant was also found to be associated with HDL cholesterol, LDL cholesterol, and TC/HDL. Differences in the G allele frequency (0.22 vs. 0.14) and effects on TG (0.14 vs. 0.30) [7] were found between Chinese and Europeans, suggesting a higher risk allele frequency and weaker effect in Chinese population. The *APO(A1/C3/A4/A5)* cluster encodes important regulators of fasting lipids, and there is considerable evidence suggesting that variants in this region are associated with altered lipid metabolism [20], [21]. Fine mapping in this region may help us to find the functional variant.

**Figure 1:**
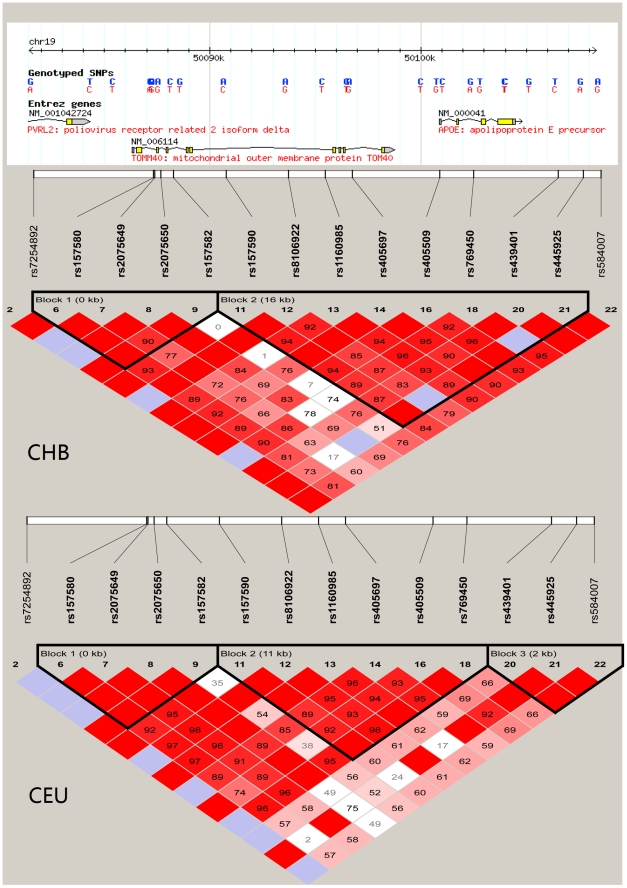
Linkage disequilibrium (LD) structure of the SNPS. Linkage disequilibrium (LD) structure of SNPs near the *TOMM40-APOE* locus in HapMap CHB and HapMap CEU. The scheme of the representation of LD is according to the default setting of Haploview Software.

Another similar case is rs157580 in *TOMM4-APOE,* which encodes Apolipoprotein E, a main apoprotein of the chylomicron, essential for the normal catabolism of triglyceride-rich lipoprotein constituents. We found the A allele of rs157580 was associated with decreased LDL, TC and increased TG in Chinese, which was different from European population (A allele was associated with increased LDL, TC and TG) (Table 4 and Table S1) [4]. Two other SNPs rs4420638 and rs439401 in this region were reported to be associated with blood lipid profile in both Europeans and Chinese [4], [12], [13], and rs439401 (not included in this study) also showed different effect direction on LDL and TC between the two ethnic groups. Given the different allele frequencies (A allele of rs157580, 0.44 vs. 0.67) and different linkage equilibrium patterns of this region (Figure 1) between Chinese and Europeans, these discordant results across ethnic groups could be explained by different linkage patterns between the causal variants and the tag SNPs that were studied. Nevertheless, these results confirmed the involvement of variants of this gene cluster in the lipid metabolism.

We found variant in *ABCA1* associated with TC, TG and HDL. The association with TG was newly identified and retained significant after adjusting by other lipid traits (data not shown), suggesting variant in *ABCA1* gene played a wider effect on blood lipid profile in the Chinese population. Recently, Acuna-Alonzo et al [22] reported a functional *ABCA1* gene variant exclusive to Native American and descent populations is associated with low HDL cholesterol levels and shows evidence of positive selection. These findings suggest the importance of *ABCA1* genetic variants in lipid metabolism.

We also found variant in *DOCK7* associated with TC and TG, variant in *TIMD4-HAVCR1* associated with TC, TG, LDL and TC/HDL, variant in *TRIB1* associated with TC, TG, LDL, and TC/HDL, with similar effects and same effect directions on blood lipid traits as previous studies in Europeans [4], [7], [9], [12]. Our study confirmed that these loci are implicated in lipid metabolism in the Chinese as well as the European populations.

The *FADS1/2/3* cluster locates on 11q12 encoding fatty acid desaturases, which convert polyunsaturated fatty acids into cell signaling metabolites and are functionally involved in lipid metabolism. Previous European studies found variants in the *FADS1/2/3* cluster to be associated with plasma concentrations of TG, TC, HDL cholesterol and LDL cholesterol [4], [7], [9], [12]. We found rs174546 in this locus marginally associated with TG (*P*∼0.01) in Chinese population, with same effect direction to that in Europeans. This result is consistent with another East Asian study, which reported a SNP in FADS1/2 is associated with TG in Japanese and associated with LDL in Mongolian. The linkage disequilibrium patterns of this region in the Chinese population somewhat differ with Europeans. For instance, the LD value between rs174546 and rs174570 is much higher in Chinese (D' = 1.0, r^2^ = 1.0, HapMap, CHB) than in Europeans (D' = 1.0, r^2^ = 0.32, HapMap, CEU). The different linkage disequilibrium pattern may therefore explain different association profile across the two ethnic groups.

Rs2338104 in *MMAB-MVK* was reported to be associated with HDL cholesterol in studies by Willer [11] and Kathiresan [7], but in our study this variant showed no association with HDL cholesterol, which is consistent with a large scale Japanese study [14]. Similarly, variants in *NR1H3*, *LIPG*, *DNAH11*, *HNF1A* and *MAFB* didn't show significant association with blood lipid traits in our study. It is not necessarily the case that these loci do not influence lipid phenotypes in Chinese. One possible reason is that because of the modest effect sizes of the individual genetic variants on lipid traits our sample size is not enough to detect the association. Another possible reason is the different linkage disequilibrium pattern in Europeans and East Asian population. It is possible that these genes may influence lipid levels through other polymorphisms in East Asian populations. Fine mapping these regions by deep sequencing or additional screening of dense arrays would be needed to reveal association between these genes and lipid levels in the Chinese population.

In conclusion, we successfully replicated association between 7 loci and plasma lipid concentrations in the Chinese population. Our study confirmed the implication of *APO(A1/C3/A4/A5), TOMM40-APOE, ABCA1, DOCK7, TIMD4-HAVCR1, TRIB1* and *FADS1/2* in plasma lipid and lipoprotein concentrations in Chinese population.

## Supporting Information

Table S1 **Comparison of the effect directions between Chinese and Europeans.**
(DOCX)
