# Supplementary material for: Association of Genetic Loci with Blood Lipids in the Chinese Population
Source: PLoS One. 2011 Nov 3;6(11):e27305. doi: 10.1371/journal.pone.0027305 (PMC3207848; doi:10.1371/journal.pone.0027305)
Supplement: Table S1 — Comparison of the effect directions between Chinese and Europeans. (DOCX) [file pone.0027305.s001.docx]

| Table S1 Comparison of the effect directions between Chinese and Europeans | | | | | | | |  |  |  |  |  |  |  |  |  |  |  |  |  |  |
| --- | --- | --- | --- | --- | --- | --- | --- | --- | --- | --- | --- | --- | --- | --- | --- | --- | --- | --- | --- | --- | --- |
|  |  |  |  |  |  |  | TC | | |  | log(TG) | | |  | HDL | | |  | LDL | | |
| SNP | Chr. | Position(Mb) | Nearest gene(s) | Minor  Allele | CN_Freq^a^ | EU_Freq^b^ | EU_Dir^c^ | CN_Dir^c^ | Ref. |  | EU_Dir^c^ | CN_Dir^c^ | Ref. |  | EU_Dir^c^ | CN_Dir^c^ | Ref. |  | EU_Dir^c^ | CN_Dir^c^ | Ref. |
| rs10889353 | 1 | 62.89 | *DOCK7* | C | 0.19 | 0.32 | - | **-** | 4 |  | - | \ | 4 |  | - | \ | 4 |  | - | \ | 4 |
| rs1501908 | 5 | 156.33 | *TIMD4-HAVCR1* | G | 0.27 | 0.37 | \ | - | 7 |  | \ | - | 7 |  | \ | \ | 7 |  | - | - | 7 |
| rs2954029 | 8 | 126.56 | *TRIB1* | A | 0.42 | 0.53 | \ | + | 7,12 |  | + | + | 12 |  | \ | \ | 7,12 |  | \ | - | 7,12 |
| rs1883025 | 9 | 106.7 | *ABCA1* | T | 0.22 | 0.25 | \ | - | 7,12 |  | \ | - | 7,12 |  | - | - | 12 |  | \ | \ | 7,12 |
| rs964184 | 11 | 116.15 | *APO(A1/C3/A4/A5)* | G | 0.22 | 0.13 | \ | \ | 7,12 |  | + | + | 12 |  | - | - | 7,12 |  | \ | - | 7,12 |
| rs174546 | 11 | 61.33 | *FADS1-FADS2* | T | 0.41 | 0.34 | \ | \ | 9,12 |  | + | + | 12 |  | - | \ | 9,12 |  | \ | \ | 9,12 |
| rs2338104 | 12 | 108.38 | *MMAB-MVK* | G | 0.37 | 0.55 | \ | \ | 7 |  | \ | \ | 7 |  | + | \ | 7 |  | \ | \ | 7 |
| rs2650000 | 12 | 119.87 | *HNF1A* | A | 0.47 | 0.36 | \ | \ | 7 |  | \ | \ | 7 |  | \ | \ | 7 |  | + | \ | 7 |
| rs157580 | 19 | 50.09 | *TOMM40-APOE* | A | 0.44 | 0.67 | + | - | 4 |  | + | \ | 4 |  | - | \ | 4 |  | + | - | 4 |
| rs6102059 | 20 | 38.66 | *MAFB* | C | 0.44 | 0.68 | *\* | *\* | 7 |  | \ | \ | 7 |  | \ | \ | 7 |  | - | \ | 7 |
| a. Allele frequency estimated from our Chinese population | | | | | | |  |  |  |  |  |  |  |  |  |  |  |  |  |  |  |
| b. Allele frequency in Europeans from references | | | | | | | | | | | |  |  |  |  |  |  |  |  |  |  |
| c. Effect directions in Europeans and Chinese. "+" represents that the minor allele is associated with increase of the trait. "-" represents that the minor allele is associated with decrease of the trait. "\" represents no available data or negative association. | | | | | | | | | | | | | | | | | | | | | |
|  | | | | | | |  |  |  |  |  |  |  |  |  |  |  |  |  |  |  |
|  | | | | | |  |  |  |  |  |  |  |  |  |  |  |  |  |  |  |  |
